# The GSH responsive indocyanine green loaded PD-1 inhibitory polypeptide AUNP12 modified MOF nanoparticles for photothermal and immunotherapy of melanoma

**DOI:** 10.3389/fbioe.2023.1294074

**Published:** 2023-10-19

**Authors:** Ying Hao, Tailuo Liu, Hao Zhou, Jinrong Peng, Ka Li, Yuwen Chen

**Affiliations:** ^1^ West China Hospital, Sichuan University/West China School of Nursing, Sichuan University, Chengdu, China; ^2^ Laboratory of Heart Valve Disease, West China Hospital, Sichuan University, Chengdu, China; ^3^ Department of Cardiology, West China Hospital, Sichuan University, Chengdu, China; ^4^ State Key Laboratory of Biotherapy and Cancer Center, West China Hospital, Sichuan University, and Collaborative Innovation Center of Biotherapy, Chengdu, China

**Keywords:** ICG, AUNP12, MOF nanoparticles, photothermal therapy, immunotherapy

## Abstract

**Introduction:** Photothermal therapy (PTT) holds significant potential for the treatment of malignant tumors. However, conventional single PTT often struggles to effectively inhibit tumor metastasis and recurrence. In this study, we constructed a MOF nanoparticle with a synergistic therapeutic effect combining photothermal and immunotherapy, enabling selective blocking of the PD-1/PD-L1 pathway within the tumor microenvironment.

**Methods:** Firstly, MOF nanoparticles were synthesized using NH_2_-TPDC as ligands and Zr^+4^ as metal ions. Subsequently, NH_2_ was modified to N_3_ via azide transfer reagents. Through a copper free catalytic click chemical reaction, the PD-1/PD-L1 blocking agent AUNP-12 functionalized with disulfide bonds of DBCO was covalently introduced into MOF nanoparticles which were then loaded with the photothermal agent indocyanine green (ICG) to successfully obtain uniformly sized and stable ICG-MOF-SS-AUNP12 nanoparticles.

**Results and discussion:** ICG-MOF-SS-AUNP12 exhibited GSH-triggered release of PD-1/PD-L1 blockers while demonstrating potent photothermal effects capable of efficiently killing tumor cells. Under 808 nm near-infrared (NIR) irradiation, ICG-MOF-SS-AUNP12 effectively promoted the maturation of DC cells and activated immune responses. This study presents a novel method for constructing MOF-based nanodrugs and offers new possibilities for the synergistic treatment of tumors involving photothermal combined with immunotherapy.

## 1 Introduction

Photothermal therapy (PTT), as a non-invasive cancer treatment strategy, utilizes external illuminants to convert light energy into heat for the purpose of eradicating tumor cells (Hao et al., 2017; Hao et al., 2020a; Yin et al., 2023). The suppression of tumor metastasis and recurrence remains a formidable challenge for single PTT (Chen et al., 2019; Chang et al., 2021). Although thermal ablation of tumor tissue can generate endogenous antigens, which can be presented to lymph nodes through antigen-presenting cells (APCs) and activate cytotoxic T cell-mediated cellular immunity, the tumor-associated antigens induced by PTT are insufficient for effective APC presentation and immune response activation (Yu et al., 2021; Huang et al., 2023). Therefore, in order to overcome the limitations of photothermal therapy (PTT) and enhance tumor immunotherapy, it is imperative to develop novel nanomedicines that can synergistically combine PTT with immunotherapy for effective tumor treatment.

The utilization of the human immune system in immunotherapy to combat diseases has garnered significant attention, particularly in the field of tumor immunotherapy (Tan et al., 2020). The immune checkpoint blockade represents an exceptionally efficacious immunotherapeutic approach for the treatment of tumors (Li et al., 2018; Zhang et al., 2022). Among them, programmed cell death protein-1 (PD-1)/programmed cell death ligand 1 (PD-L1) are commonly employed in tumor immunotherapy (Hu et al., 2021). PD-1 is a cell surface receptor expressed in activated B and T cells; however, the tumor microenvironment could induce infiltrating T cells to overexpress PD-1 molecules. These molecules bind to the ligand PD-L1 on the surface of tumor cells, transmitting immunosuppressive signals that inhibit T cell activity, and lead to immune escape of tumor cells (Leone and Emens, 2018; Wong et al., 2021). Therefore, blocking the interaction between PD-1 and PD-L1 on the surface of tumor cells is an essential strategy for preventing immune escape, thus activating T cell activity to inhibit proliferation and metastasis of tumors (Liu et al., 2021). Currently, there exist several monoclonal antibodies capable of inhibiting the PD-1/PD-L1 pathway, including pembrolizumab, navuzumab, bavensia, devaluzumab and others. Nevertheless, these antibodies not only exhibit a high cost and significant inter-individual variability but also elicit substantial toxicity and immune reactions. It is noteworthy that Aurigene and Pierre Fabre have jointly developed the PD-1 inhibitory polypeptide AUNP12 (AUR-12/Aurigene-012), which exhibits specific binding to PDL-1 and effectively blocks the PD-1/PD-L1 pathway in preclinical investigations (Zhan et al., 2016). Compared with monoclonal antibody therapeutics, AUNP12 demonstrates cost-effectiveness and a favorable side effect profile. By impeding the PD-1/PD-L1 pathway, it exerts potent antitumor effects across various malignancies, thereby displaying promising prospects for broad application in tumor immunotherapy.

**FIGURE 1:**
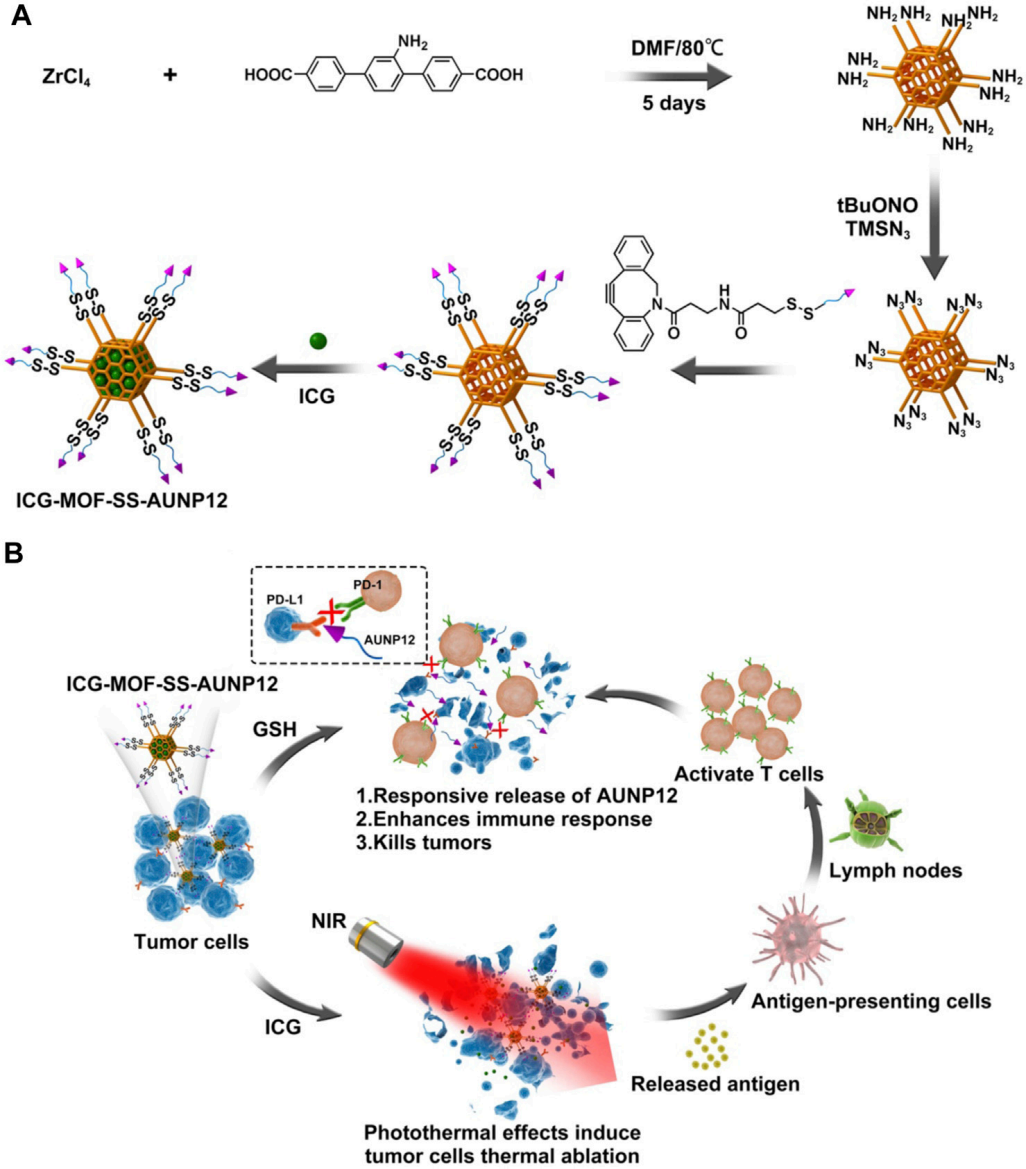
The schematic diagram of **(A)** the construction and **(B)** the anti-tumor activity of ICG-MOF-SS-AUNP12 nanoparticles for synergistic photothermal and immunotherapy.

Metal Organic Framework (MOF) is an organic inorganic hybrid material with micro/mesoporous formed by self-assembling organic ligands and inorganic metal ions or clusters through coordination bonds (Ding et al., 2022). The MOF nanoparticles exhibit several advantages over conventional drug delivery carriers, including their cost-effectiveness, excellent biodegradability, high drug loading capacity, and facile functionalization (Zhuang et al., 2017; Feng et al., 2019). Currently, they have found extensive applications in the fields of drug delivery, biosensing, and bioimaging (Wang et al., 2022). Copper, iron, zinc, and zirconium are commonly employed as metal ions for constructing MOF structures with specific organic ligands. Notably, zirconium ions can react with ligands to generate MOF carriers featuring amino groups on their surfaces (Cavka et al., 2008). In comparison with other MOF carriers, zirconium-based MOF carriers exhibit remarkable biocompatibility, thermal stability, and chemical stability. Moreover, the carrier can be easily modified through amino groups. Chen et al. functionalized the surface amino groups of MOF carriers with azide and employed click chemistry to attach nucleic acid aptamers onto the MOF surface. The loading performance and release behavior were investigated by incorporating anti-tumor drugs doxorubicin (Chen et al., 2017). In this study, we modified the surface of MOF by introducing a tumor microenvironment-responsive PD-1 inhibitory polypeptide AUNP12 with a disulfide bond through click chemistry. The photothermal agent indocyanine green (ICG) (Jiang et al., 2021) was also loaded into AUNP12 modified nanodrug (ICG-MOF-SS-AUNP12) for melanoma treatment (Figure 1). The use of MOF nanocarrier improved the stability of ICG and enabled photothermal therapy to kill tumor cells effectively. Additionally, the high concentrations of glutathione (GSH) in tumor tissues responsively broke the disulfide bond, leading to the release of AUNP12 at the tumor sites and achieving synergistic photothermal and immunotherapy for melanoma. This study further demonstrates the promising application of MOF nanocarrier in tumor therapy.

## 2 Materials and methods

### 2.1 Materials

2,5-Dibromoaniline, 4-methoxycarbonylphenylboronic acid, cesium fluoride, palladium acetate, triphenylphosphine, tert butyl nitrite, and azide trimethylsilane were purchased from Shanghai Titanchem Co., Ltd. N-hydroxysuccinimide, 1-(3-dimethylaminopropyl)-3-ethylcarbodiimide hydrochloride, zirconium chloride, indocyanine green were purchased from Sigma Aldrich Company. PD-L1 inhibitory polypeptide AUNP-12 (sequence: H-SNTSESFKF (H-SNTSESF) RVTQLAPKA QIKE-NH_2_) was purchased from Anhui Qiangyao Peptide Technology Co., Ltd.

### 2.2 Preparation and characterization of aminotriphenylenedicarboxylic acid (NH_2_-TPDC)

**FIGURE 2:**
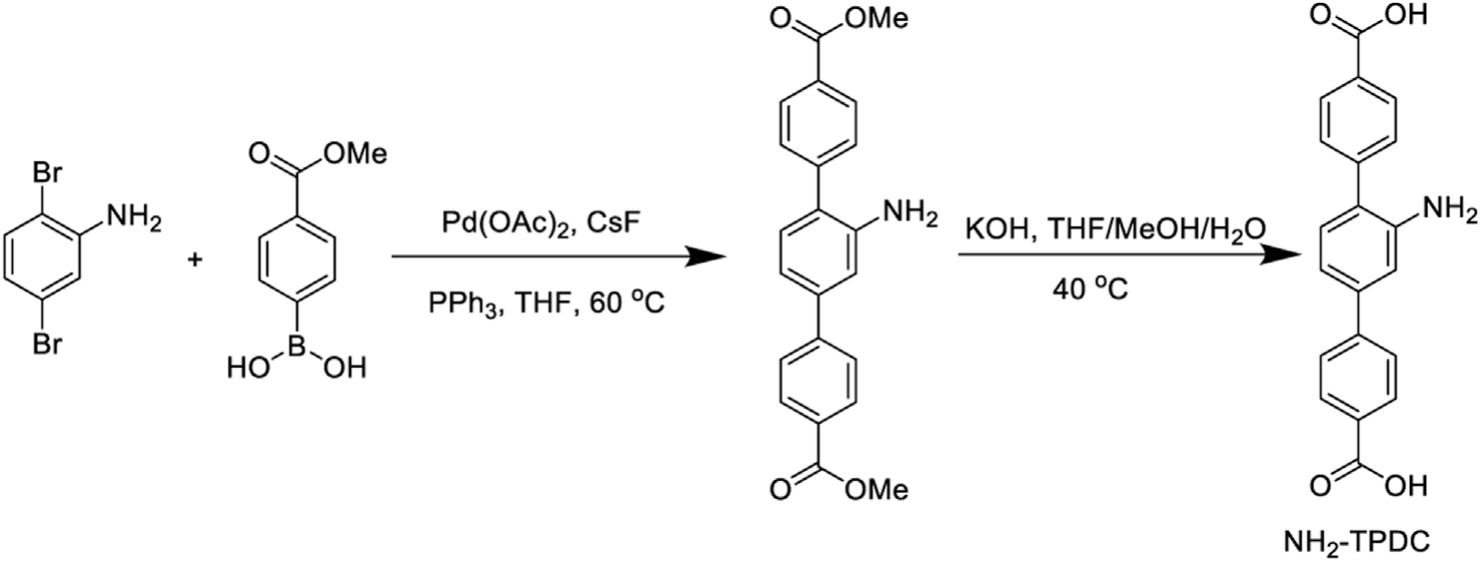
The synthesis route of NH_2_-TPDC.

502 mg of 2,5-dibromoaniline, 1.44 g of 4-methoxycarbonylphenylboronic acid, and 2 g of cesium fluoride were dissolved in 40 mL of anhydrous tetrahydrofuran (THF), then added 150 mg palladium acetate (Pd(OAc)_2_) and 400 mg triphenylphosphine (PPh3), and stir under nitrogen at 50^°^C for 48 h. Subsequently, added water and extracted with ethyl acetate 3 times, then combined the organic phase, and washed with water 3 times, dried with anhydrous sodium sulfate, and purified with a silica gel column to obtain yellow solid powder. Afterward, the yellow solid powder was dissolved in 2 mL of anhydrous tetrahydrofuran, methanol (MeOH), and 1 M potassium hydroxide (KOH) to react at 40^°^C overnight, and the yellow green solid powder NH_2_-TPDC was obtained by centrifugal washing. The synthesis process is shown in Figure 2. The product structure was characterized by nuclear magnetic resonance (NMR) spectroscopy, mass spectrometry, and infrared (IR) spectroscopy.

### 2.3 Preparation and characterization of functionalized polypeptide DBCO-SS-AUNP12

**FIGURE 3:**
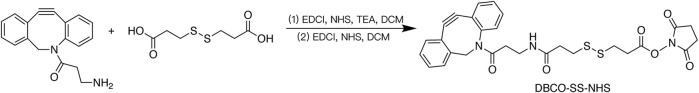
The synthesis route of DBCO-SS-NHS.

Dissolved 7 mg DBCO-NH_2_ and 3,3′-dithiodipropionic acid in 10 mL dichloromethane (DCM), and added 12 mg N-hydroxysuccinimide (NHS) and 19 mg 1-(3-dimethylaminopropyl)-3-ethylcarbodiimide hydrochloride (EDCI) in an ice bath, after reacted for 1 h, continue the reaction at room temperature overnight. The reaction is monitored by thin layer chromatography (TLC), then washed and dried to obtain a white solid. Dissolved the white solid in 5 mL DCM, added 12 mg NHS and 19 mg EDCI at room temperature to react overnight, and monitored the reaction completely with TLC. After washing and column chromatography purification, a colorless oily substance DBCO-SS-NHS was obtained. The synthesis process is shown in Figure 3.

Finally, a PBS solution of 0.1 M AUNP12 was dropwise added into DMF solution of DBCO-SS-NHS (DBCO-NHS). The amino groups on AUNP12 were connected to DBCO-SS-NHS (DBCO-NHS) through a condensation reaction. After reacting overnight, DBCO-SS-AUNP12 (DBCO-AUNP12) was obtained by dialysis and freeze-drying.

### 2.4 Preparation and characterization of MOF-SS-AUNP12

**FIGURE 4:**
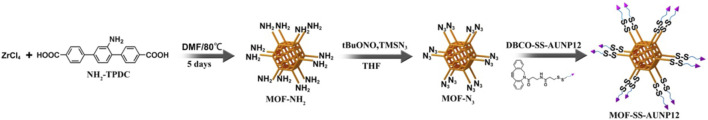
The preparation diagram of MOF-SS-AUNP12.

In this project, zirconium chloride (ZrCl_4_) was used as the metal ion, and NH_2_-TPDC as the ligand to prepare MOF material. In detail, the 12 mg NH_2_-TPDC and 8.4 mg ZrCl_4_ were dissolved in DMF, and an appropriate amount of acetic acid was added to stirred at 80°C, following reacting for 5 days, centrifuged and washed with DMF to obtain MOF. Subsequently, 1 mg of MOF was dispersed in 2 mL of acetonitrile, and an appropriate amount of tert butyl nitrite (t-BuONO) and azide trimethylsilane (TMSN_3_) were added for stirring overnight, and centrifugated to get N_3_-MOF. The N_3_-MOF and DBCO-SS-AUNP12 were stirred at room temperature for 3 h, and centrifuged and washed with DMF to obtain MOF-SS-AUNP12. The preparation process is shown in Figure 4. The MOF nanocarrier was characterized by particle size and zeta potential.

### 2.5 Preparation and characterization of ICG-MOF-SS-AUNP12

**FIGURE 5:**
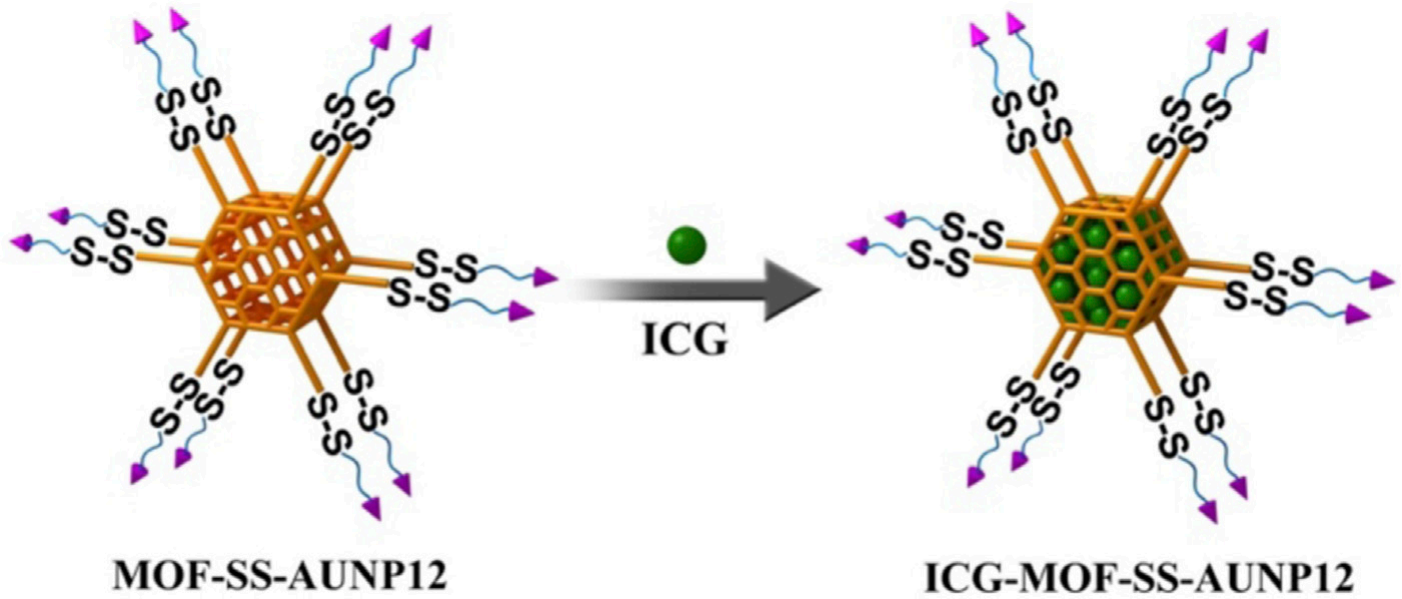
The preparation diagram of ICG-MOF-SS-AUNP12.

As shown in Figure 5, the photothermal agent ICG and MOF-SS-AUNP12 were stirred overnight in PBS solution, centrifuged, and washed with PBS to obtain nanodrug ICG-MOF-SS-AUNP12. The drug loading capacity and encapsulation efficiency of ICG were investigated via a UV spectrophotometer. The ICG-MOF-SS-AUNP12 was characterized in terms of particle size, zeta potential, TEM, and SEM. In addition, the photothermal ability of ICG-MOF-SS-AUNP12 directly affects the therapeutic effect. So we investigated the photothermal effects of ICG-MOF-SS-AUNP12 under an 808 nm NIR laser at a density of 1.5 W/cm^2^, and recorded the temperature changes through infrared thermal imaging instruments.

### 2.6 Release behavior of ICG-MOF-SS-AUNP12

The release behavior of AUNP12 in ICG-MOF-SS-AUNP12 under different concentrations of GSH (0, 2 μM, 10 mM) at 37^°^C with a shake at 100 rpm was tested (Hao et al., 2023). Samples were taken at determined time points, and the release behavior of AUNP12 at different time points was detected through high performance liquid chromatography (HPLC).

### 2.7 Biological evaluation of ICG-MOF-SS-AUNP12

In this study, via live and dead cell staining experiments, we estimated the cell inhibiting ability of ICG, AUNP12, MOF-SS-AUNP12, and ICG-MOF-SS-AUNP12, equal to 100 μg/mL of ICG and 200 μg/mL of AUNP12, on mouse melanoma cell lines B16 cells which have cultured in RPMI 1640 medium at a density of 5 × 10^4^ in a 24-well plate, with or without NIR light irradiation (1.5 W/cm^2^, 5 min). Moreover, the cytotoxicity of ICG, AUNP12, MOF-SS-AUNP12, and ICG-MOF-SS-AUNP12 was also carried on B16 cells with a density of 5 × 10^3^ in a 96-well plate through MTT methods (Hao et al., 2020b) as reported previously.

### 2.8 Immunological effects of ICG-MOF-SS-AUNP12

**FIGURE 6:**
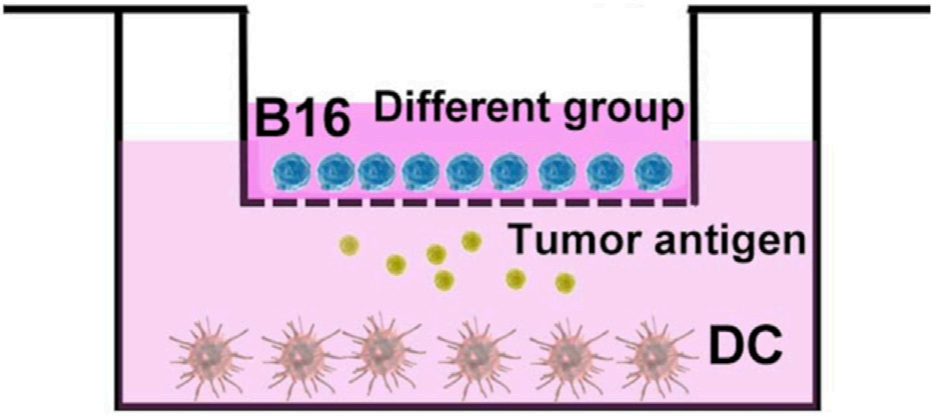
Schematic diagram of co-culture of DC cells and B16 cells.

The dendritic cells (DC cells) were extracted from mouse bone marrow, and induced maturation by adding cytokines such as granulocyte-macrophage colony stimulating factor (GM-CSF) and interleukin 4 (IL-4) *in vitro*. Subsequently, as shown in Figure 6, DC cells with a density of 2 × 10^5^ in RPMI 1640 medium were placed at the lower layer of transwell, and B16 cells with a density of 5 × 10^4^ in RPMI 1640 medium were co-cultured in transwell chamber. Then the group of AUNP12, ICG+Laser, ICG-MOF-SS-AUNP12, and ICG-MOF-SS-AUNNP12+Laser (1.5 W/cm^2^, 5 min) were added to transwell chamber. After 24 h of stimulation, the DC cells were collected and stained with fluorescently labeled antibodies CD11C-PE, CD86-APC, and CD80-FITC, and analyzed by flow cytometry.

## 3 Results and discussions

### 3.1 Preparation and characterization of NH_2_-TPDC and DBCO-SS-AUNP12

**FIGURE 7:**
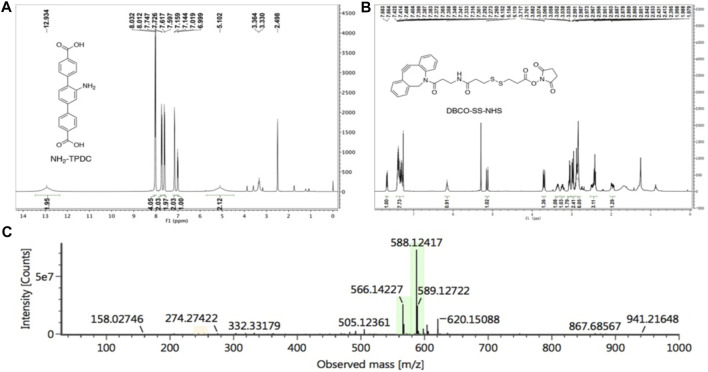
NMR spectra of **(A)** NH_2_-TPDC and **(B)** DBCO-SS-NHS, **(C)** mass spectrum of DBCO-SS-NHS.

We synthesized the MOF ligand NH_2_-TPDC by 2,5-dibromoaniline and 4-methoxycarbonylphenylboronic acid. The NMR spectra were shown in Figure 7A (1H NMR (400 MHz, DMSO) δ 12.93 (brs, 2H), 8.02 (d, J = 8.2 Hz, 4H), 7.74 (d, J = 8.2 Hz, 2H), 7.61 (d, J = 8.1 Hz, 2H), 7.15 (d, J = 5.7 Hz, 2H), 7.01 (d, J = 7.8 Hz, 1H), 5.10 (brs, 2H), the results indicated that we synthesized NH_2_-TPDC successful. DBCO-SS-NHS was synthesized from azadibenzocyclooctyne amine and 3,3′- dithiodipropionic acid, and the NMR spectra were shown in Figure 7B (^1^H NMR (400 MHz, CDCl_3_) δ 7.67 (d, J = 7.6 Hz, 1H), 7.42–7.27 (m, 8H), 6.10 (t, J = 5.2 Hz 1H), 5.14 (d, J = 7.6 Hz 1H), 3.70 (t, J = 6.4 Hz, 1H), 3.39–3.32 (m, 1H), 3.26–3.21 (m, 1H), 3.07–3.04 (m, 2H), 3.01–2.94 (m, 2H), 2.99–2.83 (m, 6H), 2.53–2.36 (m, 3H), 2.02–1.91 (m, 1H). Additionally, the calculated molecular weight of DBCO-SS-NHS (C_28_H_28_N_3_O_6_S_2_^+^) was 566.1414, and the molecular weight obtained by mass spectrometry was 566.1422 (M+H)^+^ (Figure 7C), further demonstrated the successful preparation of DBCO-SS-NHS. Subsequently, the polypeptide AUNP12, which could block the PD-1/PD-L1 pathway, was reacted with DBCO-SS-NHS to obtain a functionalized polypeptide DBCO-SS-AUNP12 for further research.

### 3.2 Preparation and characterization of ICG-MOF-SS-AUNP12

**FIGURE 8:**
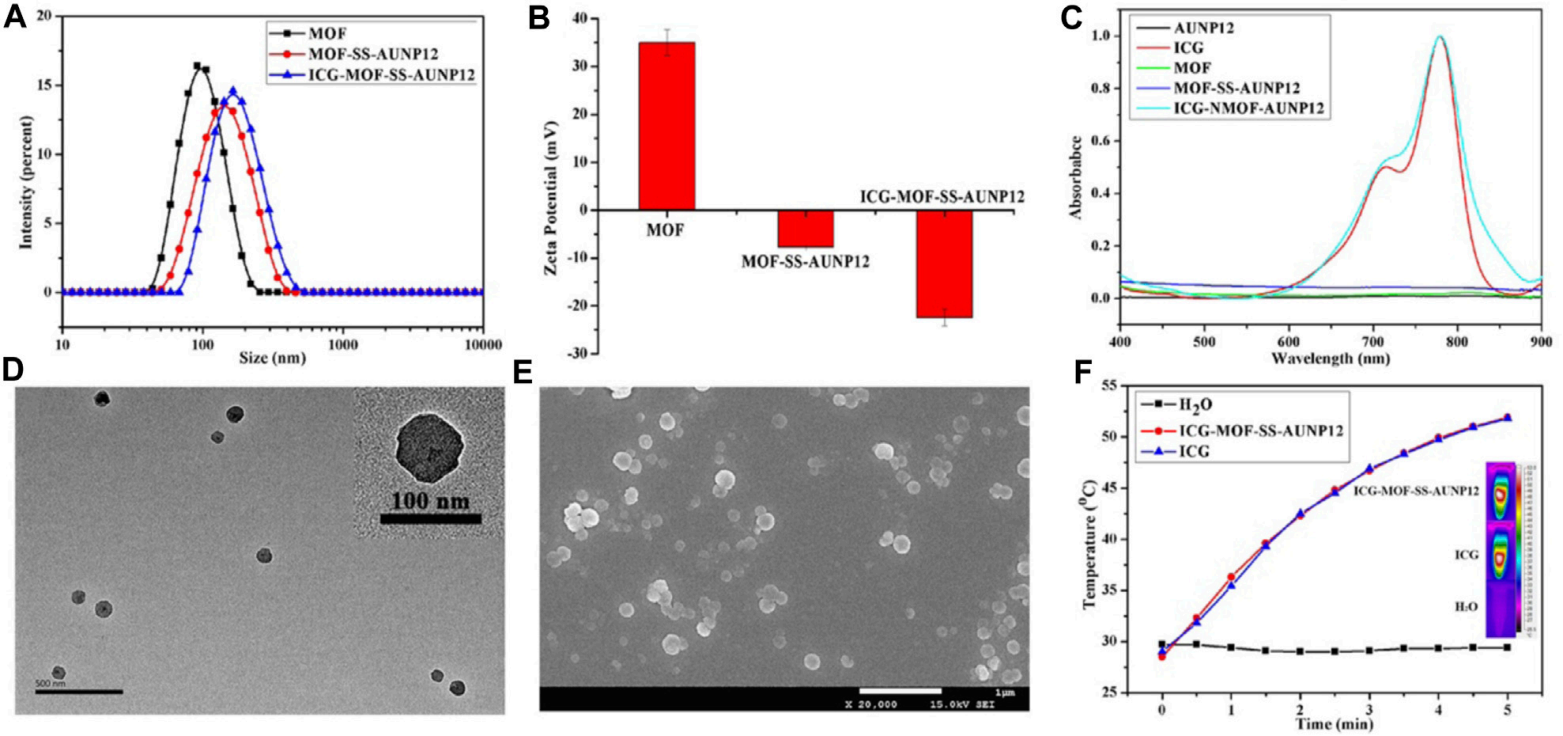
**(A)** Particle size, **(B)** Zeta potential, **(C)** UV absorption spectrum, **(D)** TEM image (scale bar: 500 nm), **(E)** SEM image (scale bar: 1 µm) and **(F)** Photothermal effect of nanoparticles.

The MOF material (particle size: 95.58 ± 0.42 nm, PDI: 0.140, zeta potential: 35 mV) was successfully synthesized using NH_2_-TPDC as the ligand and Zr^+4^ as the metal ions. Amino groups on the surface of MOF were then modified to azide. Subsequently, the functionalized polypeptide DBCO-SS-AUNP12 was connected to the MOF surface to obtain MOF-SS-AUNP12 (particle size: 123.07 ± 1.85 nm, PDI: 0.182, zeta potential: −7.74 mV). The photothermal agent ICG was loaded into the MOF to obtain ICG-MOF-SS-AUNP12 with a particle size of 152.51 ± 2.09 nm (PDI: 0.153) and a zeta potential of −22.4 mV (Figures 8A, B). The TEM and SEM images showed that ICG-MOF-SS-AUNP12 exhibited good dispersion without agglomeration (Figures 8D, E). To evaluate whether the loading of ICG affected its photothermal ability, the absorption peak of ICG-MOF-SS-AUNP12 was measured using a UV spectrophotometer (Figure 8C), which confirmed that it still maintained the characteristic absorption peak of ICG at 780 nm. Furthermore, we investigated the photothermal effect of ICG-MOF-SS-AUNP12 and free ICG via an 808 nm NIR laser and an infrared thermal imaging instrument, the results demonstrated that both samples possessed strong photothermal abilities and could rapidly reach a temperature rise up to 53^°^C within 5 min (Figure 8F), suggesting that ICG-MOF-SS-AUNP12 has great photothermal effect.

**FIGURE 9:**
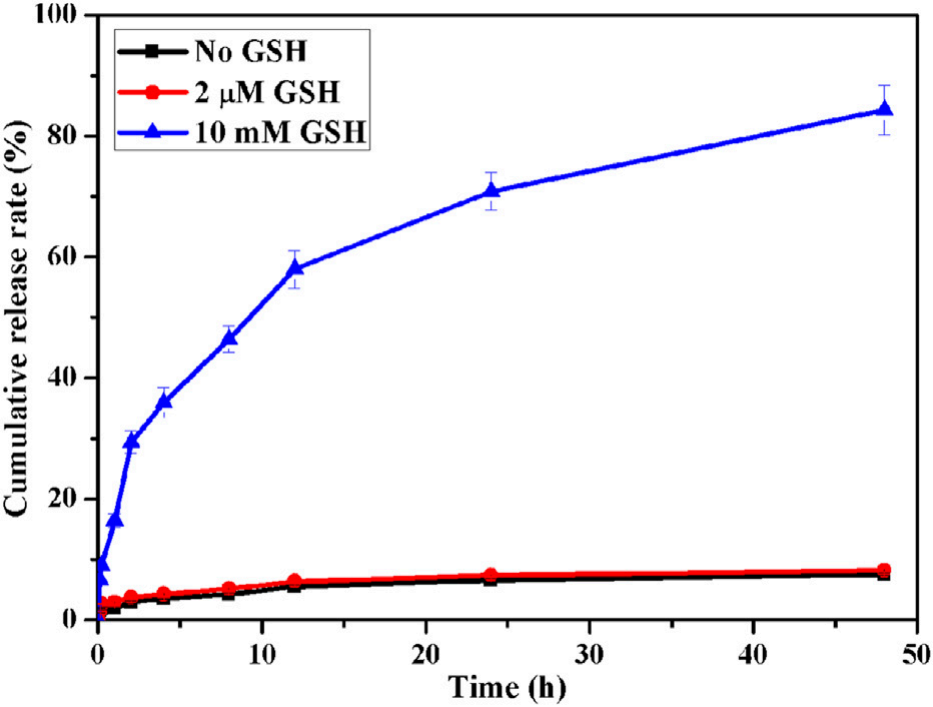
Release behavior of AUNP12 in MOF-SS-AUNP12.

In addition, we investigated the responsive release behavior of polypeptide AUNP12 in ICG-MOF-SS-AUNP12 under varying concentrations of GSH (0, 2 μM, 10 mM). As shown in Figure 9, AUNP12 exhibited negligible release without GSH or at low GSH concentration (2 µM). However, at a concentration of 10 mM, AUNP12 was released more rapidly, further demonstrating its potential for tumor microenvironment-responsive release at tumor sites.

### 3.3 Biological evaluation of ICG-MOF-SS-AUNP12

**FIGURE 10:**
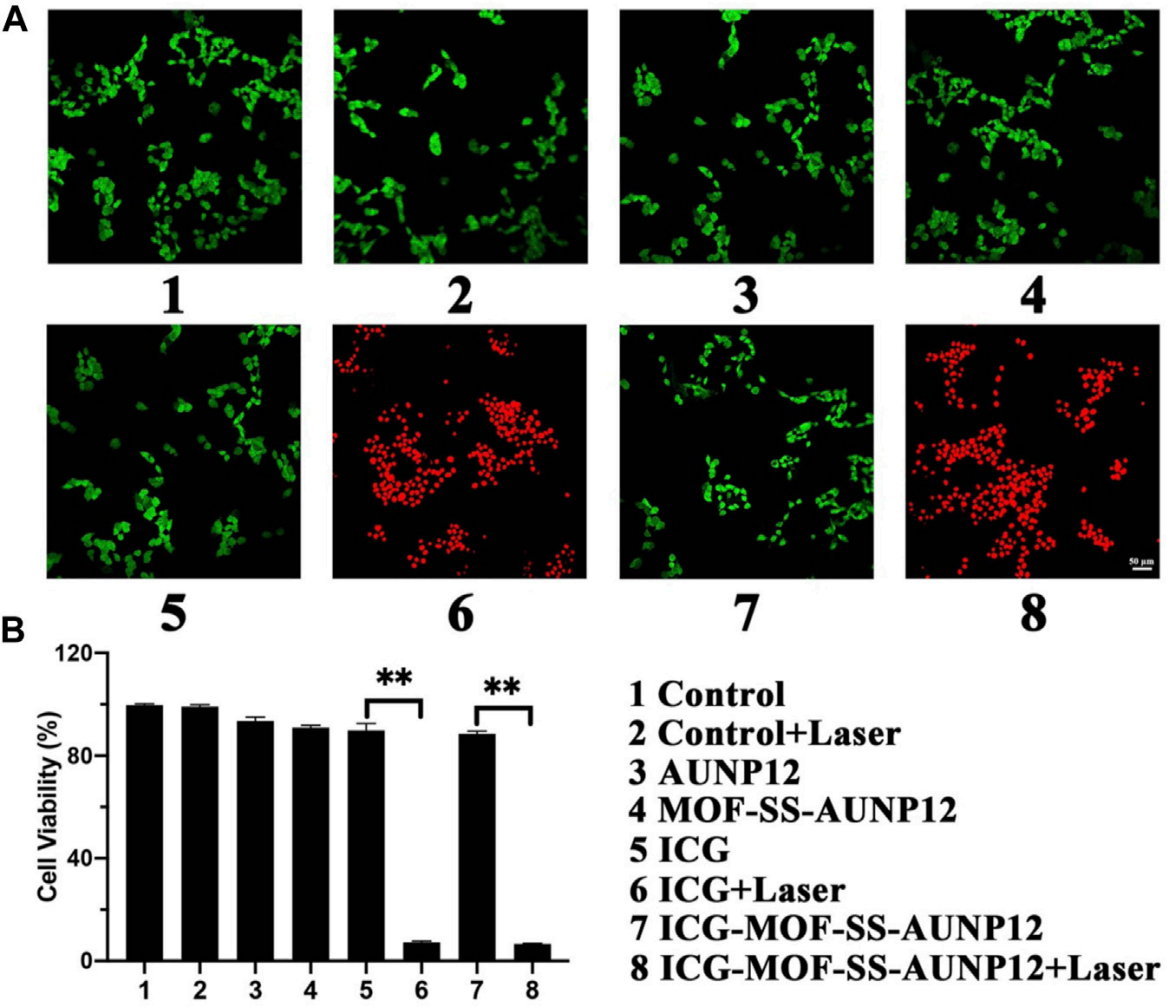
**(A)** Live and dead cell staining image (green: live cells, red: dead cells, scale: 50 µm). **(B)** Cytotoxic effect of nanodrug on B16 cells, “**” means the *p* < 0.01.

Live/dead cell staining experiments were conducted on mouse melanoma cell line B16 cells using red for dead cells and green for live cells. The results presented in Figure 10A revealed that the treatment of AUNP12, MOF-SS-AUNP12, ICG and ICG-MOF-SS-AUNP12 resulted in predominantly green-stained B16 cells indicative of cellular safety. However, when treated with the ICG-MOF-SS-AUNP12+Laser group, it showed a predominance of red-stained B16 cells, further confirming the potential therapeutic efficacy of ICG-MOF-SS-AUNP12 in tumor treatment. We also assessed the cytotoxic effects of AUNP12, MOF-SS-AUNP12, ICG, and ICG-MOF-SS-AUNP12 on B16 cells with or without an 808 nm NIR irradiation. As depicted in Figure 10B, MOF-SS-AUNP12 exhibited negligible cytotoxicity, indicating excellent biocompatibility of the MOF carrier. Furthermore, under 808 nm NIR irradiation, both the ICG group and ICG-MOF-SS-AUNP12 group demonstrated potent inhibition of B16 cells, highlighting the effective tumor-killing capability of the photothermal agent ICG.

### 3.4 Immunological effects of ICG-MOF-SS-AUNP12

**FIGURE 11:**
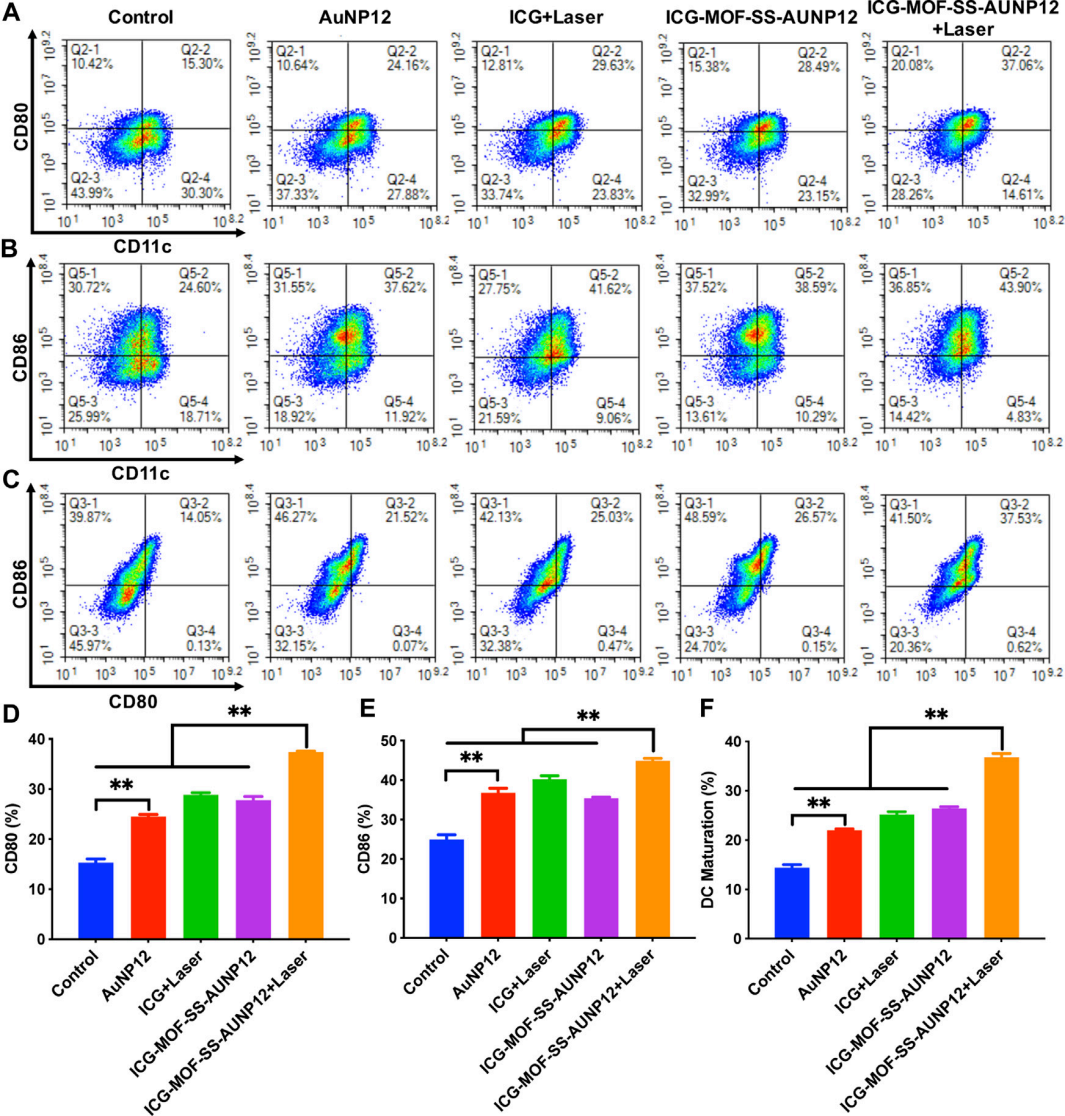
The maturity of DC Cells stimulated by different groups. **(A–C)** Expression levels of surface molecules CD80 and CD86 on DC Cells, **(D–F)** Quantification of expression levels of CD80 and CD86 on DC Cells, “**” means the *p* < 0.01.

Finally, we isolated dendritic cells (DCs) from mouse bone marrow and co-cultured them with B16 cells to investigate the ability of ICG-MOF-SS-AUNP12 to induce DC maturation. CD80 and CD86 are typical markers that indicate DC maturation. As shown in Figure 11, both polypeptide AUNP-12 and ICG-MOF-SS-AUNP12 were capable of stimulating DC maturation, resulting in a maturity rate of 21.52% and 26.57%, respectively, thereby demonstrating the polypeptide’s potential in promoting DC maturity. Furthermore, the maturity rate was observed to be 25.03% in the ICG+Laser group, indicating that simple photothermal therapy could also elicit immune responses. Notably, the ICG-MOF-SS-AUNP12+Laser group exhibited the highest maturity rate (37.53%), suggesting that photothermal effects can enhance immunotherapy effectiveness significantly. In summary, this study further validates the potential application of ICG-MOF-SS-AUNP12 in photothermal immune therapy.

## 4 Conclusion

In summary, we developed a GSH-responsive ICG loaded PD-1 inhibitory polypeptide AUNP12 modified MOF nanoparticles for achieving synergistic photothermal and immunotherapy in melanoma treatment. The ICG-MOF-SS-AUNP12 exhibits potent photothermal effects for tumor cell ablation while intelligently releasing PD-1 inhibitory polypeptide to enhance DC cell maturation. This study presents a novel approach towards the development of intelligent nanomedicine with potential applications in the synergistic treatment of melanoma.

## Data Availability

The original contributions presented in the study are included in the article/supplementary material, further inquiries can be directed to the corresponding author.
